# Creating inclusive classrooms by engaging STEM faculty in culturally responsive teaching workshops

**DOI:** 10.1186/s40594-020-00230-7

**Published:** 2020-07-01

**Authors:** Erin Sanders O’Leary, Casey Shapiro, Shannon Toma, Hannah Whang Sayson, Marc Levis-Fitzgerald, Tracy Johnson, Victoria L. Sork

**Affiliations:** 1 Center for Education Innovation and Learning in the Sciences, Divisions of Life and Physical Sciences, University of California Los Angeles (UCLA), Los Angeles, CA USA; 2 Center for Educational Assessment, Center for the Advancement of Teaching, Division of Undergraduate Education, University of California Los Angeles (UCLA), Los Angeles, CA USA; 3 Department of Molecular, Cell and Developmental Biology Department, University of California Los Angeles (UCLA), Los Angeles, CA USA; 4 Department of Ecology and Evolutionary Biology, University of California Los Angeles (UCLA), Los Angeles, CA USA; 5 Division of Life Sciences, UCLA College, University of California Los Angeles (UCLA), Department Box 951438, Los Angeles, CA 90095-1438 USA

**Keywords:** Inclusive education, Teaching practices, Culturally responsive pedagogy, Classroom climate, Equitable learning environment, Faculty development

## Abstract

**Background:**

As higher education institutions strive to effectively support an increasingly diverse student body, they will be called upon to provide their faculty with tools to teach more inclusively, especially in science, technology, engineering, and mathematics (STEM) classrooms where recruitment and retention of students from underrepresented and disadvantaged groups present long-standing challenges. Pedagogical training approaches to creating inclusive classrooms involve interventions that raise awareness of student and instructor social identities and explore barriers to learning, such as implicit bias, microaggressions, stereotype threat, and fixed mindset. Such efforts should focus on embracing diversity as an asset leveraged to benefit all students in their learning. In this paper, we describe the impact of multiday, off-campus immersion workshops designed to impart faculty with these tools. Based on analysis of workshop participant data, we report the resulting changes in faculty knowledge of factors affecting classroom climate and student success in STEM, attitudes about students, and motivation to adopt new teaching practices aimed at fostering equitable and culturally responsive learning environments.

**Results:**

Key findings indicate that attendees (1) increased their knowledge of social identities and the barriers to learning in STEM classrooms, particularly those faced by students from underrepresented groups in STEM or socioeconomically challenged backgrounds; (2) changed their attitudes about students’ abilities as science majors, shifting away from a fixed-mindset perspective in which characteristics, such as intelligence, are perceived as innate and unalterable; and (3) modified their teaching approaches to promote inclusivity and cultural responsiveness.

**Conclusion:**

Faculty members, who are linchpins in the evolution of college classrooms into settings that provide students with equitable opportunities to succeed academically in STEM, can benefit from participating in immersion workshops structured to support their awareness of issues affecting classroom culture related to race/ethnicity, LGBTQ status, religious affiliation, ability, socioeconomic status, and other social identities that contribute to disparities in STEM achievement and persistence.

## Background

Postsecondary careers in science, technology, engineering, and mathematics (STEM) disciplines show conspicuous underrepresentation of Black/African American, LatinX/Hispanic, American Indian, and Alaskan Native individuals, as well as women, compared to nationwide demographics (U.S. Census Bureau, 2018; U.S. Department of Education, 2018). This disparity is also apparent in U.S. colleges and universities, which should provide pathways for entry into the STEM workforce, thus calling for enhanced efforts to recruit and retain diverse students in STEM majors (National Academies of Sciences, 2016; National Academy of Sciences, 2011; President’s Council of Advisors on Science and Technology (PCAST), 2012). Critical to eliminating these systemic inequalities in higher education is a concerted effort to improve classroom instruction to be more inclusive and equity-minded such that all students have the opportunity to succeed academically, persist in their major field of study, and attain their intended degree. For all faculty, we believe that awareness of their implicit biases, a commitment to using culturally responsive pedagogy, adopting affirming attitudes about students, and a growth mindset are key components of inclusive education. For STEM faculty in particular, this inclusive approach to instruction creates a positive classroom climate that improves persistence (Cabrera, Nora, Terenzini, Pascarella, & Hagedorn, 1999), closes achievement gaps (Canning, Muenks, Green, & Murphy, 2019), and leads to equitable undergraduate student outcomes (Bauman, Bustillos, Bensimon, Christopher Brown II, & Bartee, 2005).

Across institutions, multiple student-focused strategies have been implemented to improve student success. These include summer bridge programs to broaden college access (Kallison Jr. & Stader, 2012; Murphy, Gaughan, Hume, & Moore, 2010), modernized advising practices (Soria, Laumer, Morrow, & Marttinen, 2017), cohort-based student retention programs (Matsui, Liu, & Kane, 2003; Stolle-McAllister, Domingo, & Carrillo, 2011; Toven-Lindsey, Levis-Fitzgerald, Barber, & Hasson, 2015), cocurricular resilience programs such as those offered through campus student learning centers (Duranczyk, Goff, & Opitz, 2006), learning communities (Zhao & Kuh, 2004), and social belonging interventions (Yeager et al., 2016). Many of these strategies have led to improvements in student outcomes, but they are focused largely on the student and often are run by administrative staff, separate from the faculty and instructors who deliver course content. Often neglected is the role and responsibility of STEM faculty to cultivate a learning environment in which all students have the opportunity to succeed academically (Whittaker & Montgomery, 2014). This negligence underutilizes faculty and their teaching as essential elements of student academic success while also effectually placing the burden to succeed academically solely on students. Moreover, student-level programs inevitably tend to subsample students thought to benefit most from the intervention at hand, whereas faculty-level improvements could scale to create inclusive classrooms in which all students have an opportunity to succeed.

Faculty must play an active role in supporting the academic success of all students (Bauman et al., 2005; Fairweather, 2008; Killpack & Melon, 2016). To this end, STEM educators have successfully implemented curricular strategies to improve student performance. For example, instructors who have introduced active learning into large-enrollment STEM gateway courses are reducing fail rates and making a positive difference in student performance outcomes (Freeman et al., 2014; Haak, HilleRisLambers, Pitre, & Freeman, 2011). These curricular efforts on the part of STEM faculty, however, are not sufficient to eliminate the equity gaps that persist across demographic groups (U.S. Department of Education, 2017) because these efforts do not address faculty attitudes and biases towards students’ various social identities (e.g., race/ethnicity, LGBTQ status, religious affiliation, ability, socioeconomic status) that may undermine student performance unintentionally. Importantly, the situational and social *context* of learning and classroom practices matter (Dewsbury, 2017a, 2017b), and faculty members play a significant role in establishing this *context*.

With a steady rise in enrollment among students of color, English as a second language (ESL) learners, and adult learners in US colleges and universities, as well as female students exceeding the number of male students in baccalaureate programs, the demographic profile of college students is changing in a manner that reflects the demographics of the entire country (Kanno & Varghese, 2010; U.S Department of Education, 2016). National efforts to broaden participation of students from groups historically underrepresented in higher education have helped diversify STEM majors in the last decade, particularly with respect to LatinX/Hispanic students (National Science Foundation, 2019). These demographic changes necessitate a transformation of our classrooms into learning environments that value diversity, foster inclusion, and engage students in authentic, interactive ways.

Research has shown that the distinct experiences and unique backgrounds brought to the college classroom by a diverse student body enhance the educational outcomes of all students (Gurin, Dey, Hurtado, & Gurin, 2002; Milem, Chang, & Antonio, 2005). As such, there is a need for professional development opportunities that support faculty members in embracing diversity as an asset and in becoming more culturally responsive in their teaching (Barrington, 2004; Gay, 2018; Marchesani & Adams, 1992; Powell, Cantrell, Malo-Juvera, & Correll, 2016; Prater & Devereaux, 2009; Villegas & Lucas, 2002). Inclusive pedagogy interventions, such as workshops, have been shown to help faculty be more intentional in their efforts to select content and incorporate instructional strategies that leverage the educational benefits of diverse classrooms (Booker, Merriweather, & Campbell-Whatley, 2016). As participants in these workshops, faculty become more self-aware of their own social identities and associated privileges, and also acknowledge and confront their own implicit biases (Cooper & Chattergy, 1993; Killpack & Melon, 2016).

Developing self-awareness, minding the privilege gap, and reducing implicit bias are essential components of inclusive pedagogy training (Dewsbury & Brame, 2019; Killpack & Melon, 2016; Prater & Devereaux, 2009). Privilege is a facet of identity derived from demographic characteristics (e.g., race/ethnicity, gender identity, socioeconomic status, educational background, geographic origin, and ability) and social capital, which together confer advantages that make success or sense of belonging in a discipline more likely. Many faculty members do not instinctively consider whether their own social identities and sense of belonging differ from that of students. Attending to this privilege gap, which can have a considerable impact on STEM persistence, is an important first step towards creating inclusive classrooms (Killpack & Melon, 2016; Villegas & Lucas, 2002). Even well-intentioned, equity-minded faculty members are vulnerable to implicit bias (Greenwald & Krieger, 2006), an unconscious yet habitual association with a pervasive stereotype that evokes a particular judgement or action in response to a stimulus affiliated with that stereotype (Devine, 1989). In the classroom, implicit biases may be tied to assumptions that instructors make about students based on their own limited experiences, or a lack of experiences or knowledge that counter stereotypical messaging (Ramsey, Betz, & Sekaquaptewa, 2013). Together, these biases may result in verbal or nonverbal communication that conveys an unwelcoming classroom environment and unequal expectations of students’ abilities, which adversely and disproportionately affect the performance of underserved students (McKown & Weinstein, 2008).

Inclusive pedagogy training also should provide a forum for faculty to explore barriers to student learning such as stereotype threat (Steele & Aronson, 1995), microaggressions (Nadal, Whitman, Davis, Erazo, & Davidoff, 2016; Solorzano, Ceja, & Yosso, 2001), and fixed mindset (Dweck, 1999). We believe that understanding these barriers and their adverse impacts on learning is a critical lever in changing faculty attitudes about students and motivating their commitment to adopt culturally responsive pedagogy. Stereotype threat is a form of social identity threat that occurs when the perceived competence of a group on a difficult task is thought to be low based on a stigmatized stereotype associated with members of that group (Steele & Aronson, 1995). Stereotype threat leads underserved students to feel added pressure to succeed, depleting cognitive resources needed for successful academic performance (Spencer, Logel, & Davies, 2016). Consequently, their grades, sense of belonging, and motivation to persist in STEM fields suffer (Dewsbury & Brame, 2019; Walton & Cohen, 2007; Walton & Spencer, 2009). Microaggressions are subtle verbal and nonverbal forms of discrimination, typically delivered unconsciously or unintentionally, which communicate negative, derogatory, and even hostile messages to members of marginalized social groups (Nadal et al., 2016). Microaggressions are the manifestation of implicit bias and may be based on any number of characteristics such as race/ethnicity, LGBTQ status, religious affiliation, ability, socioeconomic status, or other social identities. Research shows that microaggressions have a profound and adverse impact on members of historically disadvantaged groups and negatively impact campus climate (Solorzano et al., 2001). Finally, mindset refers to the perception of intellectual capacity and a belief as to whether intelligence is a fixed, innate quality, or a malleable characteristic that can be developed through effort and persistence (Dweck, 1999). Research shows that the achievement gap in courses taught by faculty who endorse fixed-mindset beliefs are twice as large as those in courses taught by faculty who espouse growth mindset beliefs (Canning et al., 2019). Moreover, students feel far less motivated in their performance efforts in classrooms with fixed-mindset faculty.

In this paper, we describe a two-day, off-campus immersion workshop for university faculty members, referred to hereafter as our Inclusive Excellence Workshop, which was designed to educate faculty about the substance of inclusive pedagogy relating to social identity and implicit bias, engage faculty in dialogue around the issues impeding student success in STEM classrooms, and help faculty move beyond awareness of the problems undermining student success to an internalization of their role in overcoming these problems. Specifically, the goals of the workshop were (1) to help faculty improve their knowledge of social identities and, in so doing, become more aware of their own and their students’ social identities; (2) to support faculty in their learning about the barriers to student success such as faculty attitudes, stereotype threat, microaggressions, and fixed mindset; and (3) to inspire faculty to take action to remove these barriers from their classrooms by adopting instructional strategies that enable all students to be academically successful. These goals are consistent with the outcomes of existing diversity interventions that have previously been shown to reduce gender bias among faculty (Carnes et al., 2015; Moss-Racusin et al., 2016). Here, we present a research study of 3 years worth of annual Inclusive Excellence Workshops, the results of which show the extent to which this intervention met our stated goals.

### Overview of the workshops

#### Participant characteristics

For each of 3 years (2015, 2016, and 2017), an average of 38 faculty members from 9 departments in the Division of Life Sciences and 6 departments in the Division of Physical Sciences were invited by the deans and associate deans to participate in an Inclusive Excellence Workshop. Each year, workshop organizers also invited select departmental academic advisors for undergraduates. The workshops started on a Thursday evening, continued all day Friday, and ended following a half day on Saturday.

In selecting each workshop cohort, efforts were made to balance the number of life and physical science faculty members represented. To confirm this occurred, aggregated institutional data on workshop attendees was obtained in accordance with the approved human subjects’ protocol (see Table 1 for demographic information of workshop participants by year). Life and physical science faculty were represented roughly equally at the workshop each year, with an overall average of 47% from life science departments and 49% from physical science departments. With an average of 48% women and 52% men in attendance, binary gender identity was equally divided, despite women being in the minority in both divisions. There were fewer participants from race/ethnicity groups historically underrepresented in STEM disciplines compared to White/Asian participants; however, the former group was overrepresented relative to the overall faculty population in the life and physical science divisions. Lastly, most participants were tenured or tenure-track faculty members, with full professors representing the largest group each year (57% in year 1, 51% in year 2, and 42% in year 3). However, among faculty participants, the average time of service at the institution gradually decreased over the 3-year period.

**Table 1:** Demographic characteristics of workshop participants in each cohort

	2015 (*N* = 35)^a^	2016 (*N* = 39)	2017 (*N* = 41)
Academic unit			
Life science	45.7%	48.7%	46.3%
Physical science	51.4%	46.2%	48.8%
Other	2.9%	5.1%	4.9%
Gender			
Female	42.9%	51.3%	48.8%
Male	57.1%	48.7%	51.2%
Race/ethnicity			
White/Asian participants	85.7%	89.7%	78.0%
Groups underrepresented in STEM^b^	14.3%	10.3%	9.8%
Not reported	–	–	12.2%
Position/rank			
Assistant professor	8.6%	15.4%	22.0%
Associate professor	22.9%	7.7%	17.1%
Full professor	57.1%	51.3%	41.5%
Non-tenure track teaching faculty	5.7%	17.9%	17.1%
Staff	5.7%	7.7%	2.4%
Duration of service at institution			
Average no. of years	16.71	14.75	11.03

^a^In first year, five students attended the workshop but were not included in the analyses

^b^Underrepresented STEM group members include faculty and staff who identify as Black/African American, Latinx/Hispanic, American Indian, or Alaskan Native as their race or ethnicity

All three cohorts were predominantly composed of faculty members whom the deans and associate deans considered to play an important role in undergraduate education. Each year, the workshops only had capacity for about 10% of the combined faculty in the life and physical science divisions. Keeping in mind representation across academic ranks, departments, and social identities, the workshop invitation criteria included instructors of large introductory courses and formal (e.g., chairs and vice chairs) and informal leaders involved in educational activities such as course transformation or other projects supporting pedagogical innovation. An underlying aim of these initial inclusive pedagogy interventions was to build a critical mass of individuals across the two science divisions who would benefit from the workshop, contribute to the collective workshop experience, and become inspired to lead change in their respective departments (Centola, 2013). About 60% of invitees agreed to attend, with a likely outcome that participation was skewed toward the more motivated faculty members given many individuals expressed in their email responses to the invitation that they would be attending because of interest in the opportunity to improve their teaching. Reasons for declined invitations, however, included schedule conflicts.

#### Workshop format

Each year, different facilitators, recommended by colleagues from other institutions who had attended similar workshops, were interviewed and selected to lead the Inclusive Excellence Workshops by the deans and associate deans. In the first year, four facilitators co-led the workshop, one led in the second year, and two the third year. Changing facilitators each year enabled us to experience different styles and approaches to an immersion workshop. Prior to each Inclusive Excellence Workshop, the facilitators visited the campus to meet informally with separate groups made up of faculty of all ranks, faculty of color, and students in order to identify the appropriate topics and focus areas for their respective upcoming workshops. Due to the variation in facilitators, the workshop agenda, as far as the order and emphasis of topics, varied from year to year. Initially, we were concerned that this level of variation might be a confounding variable in our analysis of the effectiveness of this intervention. However, the goals of the Inclusive Excellence Workshop remained consistent from year to year, and our research study was designed to measure the efficacy of the intervention as it aligned with these goals.

In the first year, undergraduate student representatives were also invited to attend the workshop to provide their perspective on their classroom learning environments. Based on feedback from some of the students who felt uncomfortable with the power disparity between themselves and faculty members and with the fear that their comments were construed as speaking for other students who shared their social identities, this practice was discontinued. Thus, for the next 2 years, the student perspective was obtained exclusively via the aforementioned pre-workshop meetings and then shared anonymously by the facilitators at appropriate times during the workshop.

Given that the workshops were immersive in nature, taking place off-campus and over multiple days, efforts were made to address participants’ childcare needs. Participants were allowed to bring their child(ren) along with a spouse or other caregiver or were provided resources to subsidize childcare at home for the duration of the workshop. In organizing the schedule, attention was paid to balance time spent in workshop sessions with social interaction and networking among colleagues during meals. Alone time was allocated by ensuring everyone had their own (and not shared) hotel rooms.

The Inclusive Excellence Workshops varied by year in their order of content and approach to addressing our three workshop goals, but all were organized into five sessions in the same overall timespan (see brief agendas in Table 2). During the sessions, participants explored their own social identities, particularly with respect to race/ethnicity but also others including LGBTQ status, religious affiliation, ability, and socioeconomic status, and they reflected on those of their students. Participants also discussed barriers to student learning and were introduced to tools and resources to support an action plan for adopting inclusive and culturally responsive teaching strategies upon return to campus. Each workshop session included a mix of guided, large group discussions, smaller breakout group activities, and some didactic content delivered more formally as presentations by facilitators. In addition to incorporating activities to foster interaction and participant engagement, the workshop facilitators presented an asset-based perspective of diversity and inclusion, consistent with the design principles of other successful diversity interventions (Moss-Racusin et al., 2014).

**Table 2:** Agenda for Inclusive Excellence Workshops by year with goals highlighted for each session

	2015	2016	2017
Thursday evening	Pre-survey, setting the framework, video presentation on inclusive education, and sharing of results of on-campus interviews	Pre-survey, creating the learning community, introductions, and goals	Pre-survey; introductions, value of learning names, and gender pronouns; and overview of dialogue training (4-stage model)
Session Goal^a^:	Goal #2	Goal #3	Goal #1
Friday morning	Introductions, goals, norms/ground rules, how we work, and defining terms (e.g., stereotypes, diversity, inclusion, culture, intent vs. impact)	Multicultural teaching, academic culture, multicultural life assessment, defining terms, and best practice/research-based examples for enhancing student academic success (e.g., group work, culturally responsive teaching, active learning, growth mindset)	History and research on intergroup dialogue; dialogue vs. debate vs. discussion; (stage 1) creating an environment for dialogue: communication guidelines/safe and brave spaces; and (stage 2) dialogue communication skills: active listening dyad activity, sharing of identity objects, and defining social identity
Session Goal^a^:	Goal #2	Goals #2 and #3	Goals #1 and #3
Friday afternoon	Setting expectations; listen to understand iceberg activity; presentation on culture, frames of reference, and impact on underserved students; and dynamics of group power (one up, one down)	Teaching styles and learning preferences and barriers to student success in STEM classrooms (e.g., strategies to counteract stereotype threat, respond to microaggressions, and overcome implicit biases)	(cont. stage 2) social identity experiences, impact on marginalized groups, understanding power, privilege, and oppression, and cycle of socialization; (stage 3) overcoming implicit bias: neuroscience of bias, microaggressions, empathy; and themes from on-campus interviews
Session Goal^a^:	Goals #1 and #2	Goal #2	Goals #1 and #2
Friday evening	Continuation of afternoon session	Group presentations on bias incidents in the classroom	(Stage 4) taking action/becoming an ally: responding to difficult moments in the classroom, factors affecting classroom climate, and role play activity with STEM-relevant scenarios
Session Goal^a^:	Goals #1 and #2	Goal #2	Goal #3
Saturday morning	Making connections/perspectives of persons of color; student voices/what students need to thrive; reflection and action planning for creating an environment of inclusion; and post-survey	Diversity and inclusion; Issues facing URG faculty in STEM; social identity self-awareness and implications for the classroom; and post-survey	Tools and resources, applications to integrate into teaching, action planning; and post-survey
Session Goal^a^:	Goals #1 and #3	Goals #1 and #3	Goal #3

^a^Goal #1, social identity awareness; Goal #2, understanding barriers to learning in diverse classrooms, including the impact of faculty attitudes on students; Goal #3, taking action to modify teaching practices in ways that support the success of all students

## Methods

We studied the effectiveness of the Inclusive Excellence Workshop both during and after the intervention, with data collected and analyzed for both formative and summative purposes by external evaluators each year. Data sources used in this study include surveys and informal group feedback. This study has approval for human subjects’ research (IRB# 17-001450).

### Survey data

Data were collected at three points in time. Pre- and post-workshop surveys were utilized at the beginning and end of each Inclusive Excellence Workshop, and a follow-up survey was administered a few weeks after the workshop concluded. Data collected across 3 years yielded an overall sample size (*N*) of 115 participants including 109 faculty and 6 staff members. The average response rate to the pre/post surveys was high (95.6%), while the follow-up survey was slightly lower (68.7%).

As noted previously, each workshop was led by different facilitators, and, as such, the pre- and post-survey instruments varied slightly by year with new questions added and removed based on input from the facilitators. Although the survey instruments were not identical, there were overlapping questions across different years, but not every workshop participant responded to every question. As a result, the total number of respondents (*n*) to each survey item varies and does not equal the overall sample size of 115 participants (i.e., *n* ≠ *N*). To be fully transparent in reporting the results while accounting for this variation in response rate, the frequency of responses to each survey item is reported as a percentage of the total number of respondents (*n*) and not sample size (*N*). Several weeks after the workshop, a short follow-up survey was sent to participants to request feedback on workshop effectiveness. All surveys included open- and closed-ended questions. A table mapping which items appeared in the surveys by year is provided in Additional File 1.

### Informal group feedback

To examine changes in classroom practices, we followed up with participants approximately 6 months after the Inclusive Excellence Workshop. Participants were invited to a lunch hosted by the deans and associate deans where, through a guided discussion, they were asked to reflect on their workshop experiences and, working in groups of two to three people, discuss how the workshop influenced or changed how they teach and interact with students. The groups’ comments and feedback were written down by a member of each group and given to the research team for qualitative analysis. Of the 115 workshop participants, 66 attended the follow-up luncheons in years 2 and 3 during which group comments were obtained (no documentation was collected in year 1). There was a total of 29 groups across both years. Notably, workshop participants from years 1 and 2 attended the year 2 luncheon and participants from years 2 and 3 attended the year 3 luncheon, so responses reflect a mix of participants across all 3 years.

### Measures

The workshops focused on three main goals: (1) improve social identity awareness, (2) understand barriers to learning in diverse classrooms, and (3) inspire faculty to take action to modify teaching practices. These goals became the basis for how we operationalized and measured the success of the Inclusive Excellence Workshops. To address the first goal, we used factor analysis to create a single measure of participants’ overall change in knowledge about concepts related to social identity, referred to as our “Social Identity Awareness Factor” (see Table 3). Two measures were used to address the second goal: factor analysis was used to measure change in knowledge about characteristics of a STEM classroom that can inhibit learning, referred to as our “Barriers to Student Success Factor,” and four distinct quantitative survey items were used to measure shifts in faculty attitudes about students and teaching. Finally, to address the third goal and understand what actions participants took after the workshops, we analyzed qualitative data from post-surveys and informal group feedback. Analyses, including factor modeling, significance testing, and qualitative coding, are described in detail below.

**Table 3:** Map of goals aligned with the corresponding measures and data collected during the study

Goal	Measure(s)	Data^a^
1. Improve social identity awareness	Social identity awareness factor	Pre- and post-surveys: 3-item factor
2. Understand barriers to learning in diverse classrooms	Barriers to student success factor	Pre- and post-surveys: 6-item factor
	Faculty attitudes (quantitative survey responses)	Pre- and post-surveys: 4 single-item prompts
3. Inspire action to modify teaching practices to support student success	Change in teaching practices (quantitative and qualitative survey responses)	Post-surveys: 2 single-item prompts (1 closed-ended and 1 open-ended questions)
	Feedback from informal group discussion	Open-ended discussion prompt given during follow-up meeting

^a^See Additional File 1 for a list of all survey questions and the discussion prompt

### Data analyses

To understand the impact of the workshops, we utilized a mixed-method design in which both quantitative and qualitative data were collected and analyzed as follows (Creswell, 2009).

#### Quantitative analyses

Initial analyses found pre- and post-survey items to be significantly skewed and thus not meeting the assumption of normality for conducting statistical tests of the data. Therefore, we utilized the Kruskal-Wallis Test, a rank-based nonparametric test used to assess significant differences between two or more samples, to determine if the groups had similar distributions (Breslow, 1970; Corder & Foreman, 2009). No significant differences were found by year. Therefore, the final survey sample included all 3 years of data when overlapping questions were available.

To create meaningful measures of workshop goals, exploratory factor analysis was employed using principal component analysis and a cutoff for factor loading of 0.5 for a minimum of three survey items in each latent construct. This analysis yielded two latent constructs, the “Social Identity Awareness Factor,” with factor loading values for three matching pre- and post-survey items ranging from 0.749 to 0.910, and the “Barriers to Student Success Factor,” with factor loading values for six matching pre- and post-survey items ranging from 0.522 to 0.872 (see Table 4). Reliability of each construct was measured using Cronbach’s alpha with a minimum standard cutoff of 0.6. The “Social Identity Awareness Factor” exceeded this minimum with a Cronbach’s alpha score of 0.872 for the pre-survey construct and 0.749 for the post-survey construct. Likewise, the “Barriers to Student Success Factor” had Cronbach’s alpha scores of 0.817 and 0.800 for the pre- and post-survey constructs, respectively. Table 4 presents the variables included in the constructs (e.g., relevant survey items), their factor loading values, and the Cronbach’s alpha measuring reliability for both constructs (Cortina, 1993; Cronbach, 1951; Thompson, 2004).

**Table 4:** Exploratory factor analysis of pre-survey and post-survey items

Prompt for each pre- and post-survey item (variables): Please rate your level of knowledge for each of the following topics. Likert scale: 1, not at all knowledgeable (i.e., I am unfamiliar with the topic); 2, somewhat knowledgeable (i.e., I have heard of the topic but could not readily explain it to someone else); 3, knowledgeable (i.e., I have heard of the topic and could readily explain what it means to someone else); and 4, highly knowledgeable (i.e., I understand the current research on the topic and use it to inform the way I teach)		
Social identity awareness factor (*n* = 71)		
Variables	Pre-survey factor loadings	Post-survey factor loadings
Socioeconomic status (SES)	0.910	0.859
First-generation students	0.890	0.800
Underrepresented minority (URM)	0.878	0.788
Cronbach’s alpha	0.872	0.749
Barriers to student success factor (*n* = 68)		
Variables	Pre-survey factor loadings	Post-survey factor loadings
Inclusive teaching practices	0.713	0.581
Stereotype threat	0.661	0.563
Implicit vs. explicit bias	0.522	0.596
Microaggressions	0.687	0.633
Classroom climate	0.819	0.872
Academic culture	0.525	0.581
Cronbach’s alpha	0.817	0.800

To compare mean differences in participant responses to pre- and post-survey items comprising each factor, we employed paired samples *t* tests because the distributions met the assumption of normality. To compare mean differences in participant responses to single-item survey questions addressing faculty attitudes about students and teaching, we used the Wilcoxon signed-rank tests, which is a nonparametric test used to compare two related or matched samples (Corder & Foreman, 2009).

#### Qualitative analyses

Analysis of open-ended survey questions and informal group feedback collected during follow-up luncheons followed published procedures (Creswell, 2009). Briefly, the multistep process began with an initial review and coding of participant responses by a trained qualitative research analyst (S.T., author). Qualitative responses were examined and a list of preliminary codes, or themes, was developed to capture a sense of meaningful segments of text. Subsequently, an examination of themes and text was conducted to ensure codes were relevant and succinct. Then, frequencies were calculated according to theme, and the themes were further collapsed into broader categories with those having low response frequencies (< 5%) getting omitted altogether. The process concluded when the author team reached consensus on the presented themes as accurately and concisely reflecting the sample participant responses. For this study, sample responses were pulled to illustrate how the themes corresponded to the participants self-reported experiences.

## Results

Analysis of participant responses to closed-ended survey questions, themes that emerged from open-ended survey questions, and the informal follow-up group discussions provided insights into the impact of this inclusive pedagogy intervention on faculty. Examination of responses to pre-survey items helped us understand participants’ initial levels of knowledge on topics related to diversity, attitudes about students, confidence with respect to their competency in using inclusive pedagogy, and interest in learning about and changing their teaching practices to become more culturally responsive. The post-survey asked participants to reflect on what they learned during the workshop and how their levels of knowledge, attitudes, confidence, and interest changed as a result of their participation. As related to our workshop goals, we compared pre- and post-survey items to measure changes in knowledge about concepts and classroom characteristics affecting student success in STEM classrooms, shifts in faculty attitudes about students, and the interest among faculty in modifying their teaching practices to create more inclusive and equitable learning environments. In addition, feedback from post-survey items provided insights into participants’ level of satisfaction with the workshop topics, format, and facilitator competency.

### Workshop participants are primed for intervention

This 3-year study yielded an overall sample size (*N*) of 115 workshop participants. Not every question appeared across all 3 years of the study, and not every participant responded to every question in the surveys. Thus, for each survey item, the respondent sample size (*n*) is provided, and the response frequency is reported as a percentage of the respondent sample size rather than as a percentage of the overall sample size.

The pre-workshop surveys were designed to assess the knowledge, interest, and confidence levels of participants as they checked in for the workshop. Slightly more than half of the respondents (57.6%, *n* = 111) began the Inclusive Excellence Workshop reporting they were either not at all knowledgeable or somewhat knowledgeable about the problems and challenges associated with student success in STEM. These problems include disparities in degree attainment for different groups of students, barriers to student learning such as implicit bias, stereotype threat, and microaggressions, and faculty attitudes about student ability as might be inferred from a fixed-mindset perspective. A majority of respondents were interested or very interested in learning more about the impact of social identities on classroom dynamics such as interpersonal interactions and behavior patterns (93.0%, *n* = 114) and how to modify their teaching in ways that benefit all STEM students (96.4%, *n* = 112). A little more than half (59.4%, *n* = 111) of respondents reported feeling not at all or only somewhat confident in their ability to communicate a message of sensitivity about student diversity inside or outside of the classroom.

A total of 112 workshop participants responded to open-ended pre-survey questions designed to gauge expectations and interests of workshop participants. For each question, responses were coded and grouped by theme, and then the percentage of respondents corresponding to a particular theme was reported. For example, when asked about workshop expectations, 33.0% of the 112 respondents hoped to gain awareness of or appreciation for diversity and tools to address barriers to inclusion in the classroom. Similarly, 52.7% of respondents to this same question expressed a desire to learn teaching approaches that they could use in diverse classrooms. As noted by one participant, “[I want to] learn more about my own inherent biases” and “communicate better with a wide variety of students.” Another participant stated, “I expect to inform my colleagues (at a campus level) with effective strategies to insure the success of all students regardless of background.”

### Change in knowledge of concepts and characteristics affecting inclusion in STEM classrooms

In 2016 and 2017, workshop participants were asked to describe their level of knowledge of a variety of diversity-related concepts, allowing a comparison of responses on analogous pre- and post-survey items. Factor analysis was conducted using a matched sample of pre- and post-survey responses. Two factors, the “Social Identity Awareness Factor” and the “Barriers to Student Success Factor,” had Cronbach’s alpha scores above the 0.6 cutoff value, thereby establishing the validity of these pre- and post-survey item groupings (see Table 4).

**Fig. 1:**
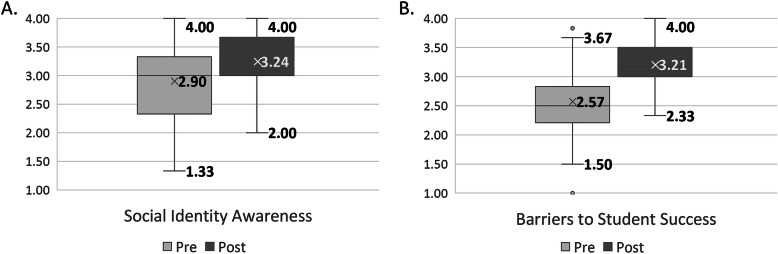
Workshop participant change in knowledge based on factor analysis of pre- and post-survey responses. **a** Awareness of social identities (*N* = 71: Mean_pre_ = 2.90, SD_pre_ = 0.66; Mean_post_ = 3.24, SD_post_ = 0.46). **b** Barriers to student success in the classroom (*N* = 68: Mean_pre_ = 2.57, SD_pre_ = 0.57; Mean_post_ = 3.21, SD_post_ = 0.41). For each factor, a paired samples *t* test indicates that the mean post-survey scores are significantly higher than the mean pre-survey scores (*p* < 0.001). Each box corresponds to the interquartile range of mean scores, the “x” corresponds to the mean score for each latent construct, the horizontal line inside each box to the median, and the dots (**b**) to outliers in the dataset. Means are derived from Likert scale values: 1, not at all knowledgeable; 2, somewhat knowledgeable; 3, knowledgeable; 4, highly knowledgeable

For the latent constructs that met the conditions for factor analysis, mean pre- and post-survey scores (based on a 4-pt Likert scale, with a score of 1 = not at all knowledgeable and a score of 4 = highly knowledgeable) were calculated by averaging the scores for all individual survey items within each construct. Paired samples *t* tests were used to test for significant differences between the mean post-workshop survey scores and the mean pre-workshop survey scores. Histograms showing the distribution of response frequencies and mean score for each survey item comprising the two factors are provided in Additional File 2. Comparison of mean pre- and post-workshop survey scores revealed a significant increase (*p* < 0.001) in self-reported knowledge of concepts presented or discussed during the workshops (Fig. 1), indicating that the intervention helped improve faculty knowledge of both their own and students’ social identities (Fig. 1a) as well as barriers to student success such as microaggressions, stereotype threat, implicit and explicit bias, a lack of inclusive teaching practices, and an unwelcoming classroom climate (Fig. 1b).

Additional results for quantitative post-workshop survey items indicate that most respondents (85.8%, *n* = 106) felt they increased their knowledge about the problems and challenges with STEM student success as a result of their participation in the workshop. Many respondents agreed (96.6%, *n* = 89) that the workshop provided new insights about enhancing student success and that they learned useful information, which will improve their teaching and interactions with students. Additionally, 83.8% of respondents (*n* = 68) indicated that their level of interest in learning more about the impact of race/ethnicity, gender, and other social identities on interpersonal interactions and behavior patterns in the science classroom increased as a result of the workshop.

### Change in faculty attitudes about students

Prior to and after the workshop, participants were asked to rate their level of agreement with a series of statements listed in Table 5 using the Likert scale: 1, strongly disagree; 2, somewhat disagree; 3, somewhat agree; and 4, strongly agree. These statements were designed to gauge faculty attitudes and opinions about students’ potential for success in STEM. This attitudinal data was collected on the pre-survey all 3 years. In 2016 and 2017, participants were asked to respond to these same statements on the post-survey. Comparison of pre- and post-survey responses using the Wilcoxon signed-rank test, a nonparametric statistical test for paired samples (Corder & Foreman, 2009), shows a small but significant shift in participants’ opinions on all four items (Table 5). Histograms showing the distribution of responses for each item are provided in Additional File 3. After the workshop, faculty were more likely to agree somewhat or strongly that it is their job to help level the playing field for students who come into their classrooms with different levels of preparedness (*Z* = 2.87, *p* < 0.01), that they should consider changing their teaching style to improve student performance (*Z* = 3.16, *p* < 0.01), and that all students are capable of success with the instructor having a role in ensuring all students have access to opportunities that promote their success (*Z* = 2.39, *p* < 0.05). The results also suggest that faculty changed their attitudes about students’ ability to succeed as science majors (*Z* = 1.99, *p* < 0.05), with a shift away from a fixed-mindset perspective about student aptitude.

**Table 5:** Descriptive statistics for survey items probing faculty attitudes about students before and after the workshop

Statements	Pre-survey			Post-survey			Difference
	*n*	Mean	SD	*n*	Mean	SD	Z
I recognize that not all students come into my classroom with the same level of preparedness; it is my job to help level the playing field.	110	3.47	0.74	70	3.74	0.53	2.87**
Some students might perform better in my class if I used a different teaching style.	113	3.41	0.66	71	3.66	0.48	3.16**
All students are capable; it is my job as their instructor to ensure that all students have equal opportunity to succeed in my class.	113	3.50	0.78	71	3.76	0.57	2.39*
Some undergraduates are not cut out to be science majors and should be encouraged to leave the major as early as possible.	112	1.88	0.86	70	1.53	0.85	1.99*

**p < 0.01, *p < 0.05 using the Wilcoxon signed-rank test

### Change in teaching practices

At the conclusion of the workshop, 83.8% of respondents (*n* = 68) to the post-survey in years 2015 and 2016 of the workshop expressed increased interest in modifying their teaching approaches in ways shown to benefit all students, especially students underrepresented and underserved in STEM. Participants responded to an open-ended question asking how they would use or apply what they learned during the workshop upon returning to campus. Across all 3 years, 53.6% of 112 survey respondents indicated an intent to incorporate new classroom practices or teaching strategies, chief among these were active and collaborative learning, grading reform, instilling a growth mindset, and setting ground rules. Many respondents (38.4%, *n* = 112) also described plans to increase communication with students as well as colleagues and teaching assistants. As noted by one participant, “I will have conversations with colleagues. I will work with the [teaching assistants] to improve discussion sections so that they can provide opportunities for interactive learning. I will encourage other students to be more inclusive, as well as educate professors who are not.”

To explore longitudinal impacts of this inclusive pedagogy intervention, participants were invited to a luncheon hosted by the deans and associate deans approximately 6 months after the workshop. During this informal follow-up meeting, participant groups comprised 2–3 participants each were formed (*N* = 29 total groups) and members of each group were asked to share some things that they had incorporated into their teaching and thought were catalyzed as a result of attending the Inclusive Excellence Workshop. Qualitative coding of 70 total responses from this group work activity generated seven themes (Table 6). Results show that more than half of the groups (55.2%) indicated they made changes to their classroom practices such as syllabus revision, namely incorporating ground rules in the syllabus and at the outset of class, and grading reform (e.g., moving away from norm-referenced grading practices such as grading on a curve). One participant stated, “I have stopped showing my grade distribution.”

**Table 6:** Themes from qualitative responses to questions asked during follow-up luncheon with workshop participants and deans

Theme	Number of responses^a^ (percent of groups^b^ indicating particular response)
Classroom practices, teaching strategies, or approaches	16 (55.2%)
Communication/interaction, sharing resources with students	16 (55.2%)
Group work, exercises, or active learning	15 (51.7%)
Awareness or respect of diversity, challenges, biases	10 (34.5%)
Student encouragement, support; availability (includes office hours)	5 (17.2%)
Confidence	4 (13.8%)
Other	4 (13.8%)

^a^*N* = 70 total responses to questions

^b^*N* = 29 groups for whom responses to questions were coded

Those participants in the follow-up meeting who reported greater communication or interaction with students (55.2% of groups, see Table 6) specifically mentioned having discussions about inclusivity and diversity issues, as well as using more gender-neutral terms in their teaching. One participant describes how the workshop “encouraged me to make myself more accessible to students by more frequently promoting my office hours and the course discussion [online] forum.” A total of 51.7% of groups mentioned incorporating group work and active learning into their teaching. Also high on the list was having greater awareness of or respect for diversity-related issues (34.5% of groups).

### Additional benefits of the workshop

In addition to evaluating the goals of the Inclusive Excellence Workshop, the post-survey and follow-up survey questions examined participant satisfaction with the intervention. After completing the workshop, a majority of survey respondents (86.7%, *n* = 98) reported feeling satisfied to very satisfied with the workshop, and most respondents (97.7%, *n* = 88) would recommend this workshop to their colleagues. Regardless of which facilitator was leading the workshop across the 3 years, respondents agreed that the workshop sessions were well facilitated and engaged participants in useful discussions (91.4%, *n* = 105) and that the facilitators created a safe environment for open and productive discussions (98.9%, *n* = 89). All respondents (100%, *n* = 89) indicated that the opportunity to meet new people in the sciences was a positive aspect of the workshop. As one respondent states, they valued “getting to know the faculty in a new intimate setting and realizing that [they] share the same goals of seeing students succeed.”

## Discussion

Findings from our study of the Inclusive Excellence Workshops illustrate that this inclusive classroom intervention can meet specific goals to expand awareness among faculty about social identities (Fig. 1a), increase their knowledge of barriers to learning (Fig. 1b), improve faculty attitudes about students (Table 5), and inspire faculty to adopt teaching strategies that support equitable and inclusive learning environments (Table 6). Before conducting the data analysis, we considered that it might be difficult to detect significant pre- and post-workshop differences because many of the participants, given their existing interest, motivation to support, and level of engagement with the teaching enterprise, would already be knowledgeable of inclusive and culturally responsive teaching practices. However, results from this study demonstrate significant learning gains among participants and also reveal what participants made actionable following this intervention.

### Creating inclusive, culturally responsive STEM classrooms

As a result of the Inclusive Excellence Workshop, instructors became more aware of various strategies to remove barriers to inclusion in their classrooms, especially for Black/African American and Latinx/Hispanic students, and to create identity-safe environments that motivate student learning. Several proposed actions were introduced during the workshop as approaches to combat stereotype threat, prevent microaggressions, and promote growth mindset. For example, instructors can foster identity-safe learning environments by infusing multicultural perspectives into the curriculum (Prater & Devereaux, 2009), exposing their students to the illegitimacy of stereotypes asserting the inferior ability of underserved students and women (Johns, Schmader, & Martens, 2005), and interrupting microaggressions when they occur (Kenny, 2014). When giving feedback to students vulnerable to stereotype threat, instructors can embrace a growth mindset whereby they accentuate their high standards while assuring students that they are all capable of meeting them (Aronson, Fried, & Good, 2002; Canning et al., 2019; Cohen, Steele, & Ross, 1999; Villegas & Lucas, 2002). Together, these actions can help to establish a welcoming learning environment in which all students feel that they are supported and valued (Davies, Spencer, & Steele, 2005).

Through their participation in the workshop, instructors came to recognize how conventional teaching practices, such as using norm-referenced grading systems (Covington, 1992; Hughes, Hurtado, & Eagan, 2014) and lecture-dominated instructional modalities (Stains et al., 2018), are associated with disparities in student success. Research studies show that interactive classroom engagement techniques, such as active learning, can reduce achievement gaps in STEM courses (Freeman et al., 2014; Haak et al., 2011). However, the situational and social *context* in which these techniques are put into practice is also important for instructors to consider (Dewsbury, 2017a, 2017b). With a better understanding of themselves and their students’ racial/ethnic and other social identities, many faculty participants reported during our informal follow-up discussions occurring months after the workshop, that they indeed took action to change their pedagogical practices. Specifically, our findings suggest that instructors are being more intentional about adopting teaching and classroom practices that support an inclusive learning environment and better serve all students in their classrooms. Notably, many of the instructors who attended the Inclusive Excellence Workshop (63.2% of 95 unique faculty) engaged in campus teaching development activities between early fall 2015 through end-of-spring term 2019 where they were presented with additional information and tools from which they might advance their knowledge and improve their teaching.

### Limitations and future research

The current study has several limitations. First, because different facilitators led the workshops each year, we did not have identical replicates of the intervention across the 3 years of the study. In addition, the order and composition of workshop activities varied from year to year (see Table 2). Despite these potential confounding factors in our analysis, our data show that faculty participation in an Inclusive Excellence Workshop leads to consistently positive outcomes with respect to our three goals, which were consistent from one year to the next. This finding suggests that goals themselves should provide the organizing framework for future immersion workshops. This goal-oriented approach is congruous with existing and effective diversity interventions (Carnes et al., 2015; Moss-Racusin et al., 2014, 2016).

Another limitation concerns the survey administration protocol. To ensure participant anonymity and encourage honest responses to survey prompts, no identifying information was collected with the surveys. Participants were asked to enter a number of their choosing (the last four-digits of a university ID number was suggested) to facilitate matching of pre/post datasets. However, some participants did not enter the same number on the pre/post surveys, consequently limiting our sample size in some cases. In addition, without identifying information, survey responses could not be linked to registrar data, which would have enabled us to directly measure the downstream effects of this inclusive pedagogy intervention on the students whom the faculty participants teach. Institutional data shows that this group of 109 faculty participants has taught a combined total of over 26,500 students across more than 1000 classes they taught since completing an Inclusive Excellence Workshop. These data demonstrate the potential for these workshops to generate a critical mass of instructors who can have a large-scale positive impact on student success (Centola, 2013). Given this promise to affect change in the academic achievement and persistence of STEM students, future efforts to study workshop outcomes necessitate collection of identifying information to make this longitudinal tracking possible.

Many faculty members reported changing their instructional approaches after the workshop (see Table 6), but previous studies show that self-report data about faculty teaching practices do not necessarily align with their actual teaching practices when verified with classroom observation data (Ebert-May et al., 2011). Conducting classroom observations of former faculty participants would improve our measure of impact on teaching practices, but resources to support and coordinate efforts to collect these additional data are needed. Additionally, conducting surveys, interviews, or focus groups with students in courses taught by faculty participants would clarify how any changes in teaching practices are affecting the classroom climate and perceived student learning experiences. A similar study reported that students felt safe, valued, and visible in courses taught by faculty who participated in diversity training and were intentional in their efforts to create an atmosphere of inclusion (Booker et al., 2016).

One final limitation of this study pertains to the data analysis. We noted that participant responses skewed positive for some of the survey items (see Table 5 and additional files 1, 2 and 3). This skew was likely an artifact of our workshop population being overly represented by faculty members who are supportive of innovative and inclusive education. Thus, we do not know the effect of this intervention on skeptical faculty who are less enthusiastic about participating in this type of inclusive pedagogy intervention or who are not currently prioritizing STEM education reform. We can comment that an Inclusive Excellence Workshop has been offered to two new cohorts since 2017. To broaden participation of faculty, including those who are not able to spend 2 days away from campus due to teaching and/or personal obligations, we have offered a workshop with a modified format, holding it on or near campus for a shorter period of time. To persuade participation of instructors who teach large-enrollment gateway courses and who were previously hesitant to take part in these workshops, invitations were extended with strong encouragement and incentives to participate, including support for substitute course instructors to make it more feasible for them to avoid teaching conflicts while attending the workshops. The evaluation of subsequent workshops is ongoing, but anecdotally, we have learned that some of the previously skeptical instructors are implementing changes in their courses. Future findings may help us determine the most optimal duration of this intervention and shed light on incentive structures needed for maximum participation.

Overall, our findings indicate that the Inclusive Excellence Workshop serves as an effective catalyst for faculty adoption of culturally responsive and inclusive teaching practices, but it is important to consider how to sustain the momentum and continue to build a coalition of instructors committed to teaching inclusively and supporting the academic success of all students. Our discipline-specific teaching and learning center in the sciences offers mini-workshops throughout the academic year, both as refreshers on social equity issues first discussed at the Inclusive Excellence Workshop and also as opportunities to turn theory into practice with training to modify teaching practices to be more culturally responsive and equity-minded in the classroom. We recognize that these longer-term supports are critical to sustaining meaningful changes in teaching practice (Kober, 2015; Prater & Devereaux, 2009).

## Conclusion

Analysis of participant data from our Inclusive Excellence Workshop shows that we were effective in our goals to raise awareness among university faculty of the social equity barriers that undermine student success in STEM, particularly those in historically underserved social identity groups, and to help faculty members understand their role in overcoming these barriers. Altogether, our findings suggest that engaging faculty in this immersion workshop had a positive impact on attitudes, increased knowledge, and motivated action to change teaching practices. We learned that critical elements to the workshops include promoting self-awareness of one’s own social identities and implicit biases, engaging in meaningful dialogue about barriers to learning as disproportionately experienced by underrepresented and disadvantaged students, and providing strategic solutions that improve classroom climate and create an asset-driven perspective of diversity in STEM. As mentioned, we did not observe significant differences in outcomes across the 3 years of the study despite each workshop being led by different facilitators. Thus, broadly speaking, one key lesson learned is that *any* intervention is better than *no* intervention when it comes to engaging faculty members in pedagogy training aimed at fostering an asset-driven perspective of STEM student diversity and building inclusive classrooms. More specifically, we found that the order of topics and composition of activities matter less than the organizing framework provided by our three goals. This takeaway provides flexibility for institutions to implement similar interventions in ways most relevant to the classroom climate or most fitting to their faculty population. Finally, we want to acknowledge that the faculty members most open to changing their teaching practices and adopting inclusive pedagogy are the ones most likely to *initially* accept an invitation to an immersion workshop like what we described here (e.g., “preaching to the choir”), an experience that empowered these instructors with more knowledge. Our data show that even among the coalition of the willing, improvements are being made. Thus, these workshops allow “the choir” to learn new songs, sing in harmony, and raise the quality of “music” that inspires others to do the same. We think that the Inclusive Excellence Workshop is creating a critical mass of instructors on board with inclusive education and who are actively improving the teaching culture. We need instructors to be fully committed to supporting the academic success of all STEM students, and immersion workshops may be a valuable tool to help them accomplish this goal.

## Supplementary information

**Additional file 1.** Survey Items by Year.

**Additional file 2.** Histograms associated with Figure 1.

**Additional file 3.** Histograms associated with Table 5.

## Data Availability

The survey instruments and datasets collected and analyzed during the study are available from the corresponding author upon reasonable request.

## Abbreviations

STEM: Science, technology, engineering, and mathematics
CURE: Course-based research experience
ESL: English as a second language
LGBTQ: Lesbian, gay, bisexual, transgender, questioning, or Queer
URG: Underrepresented group
ID: Identification
