# Supplementary material for: Creating inclusive classrooms by engaging STEM faculty in culturally responsive teaching workshops
Source: Int J STEM Educ. 2020 Jul 1;7(1):32. doi: 10.1186/s40594-020-00230-7 (PMC7326892; doi:10.1186/s40594-020-00230-7)
Supplement: Supplementary file 1 — Additional file 1. Survey Items by Year. [file 40594_2020_230_MOESM1_ESM.pdf]

Unique and Overlapping Survey Items by Year

	Closed-ended Questions	*2015*			*2016*			*2017*		
		Pre-	*Post-*	*Follow-up*	*Pre-*	*Post-*	*Follow-up*	*Pre-*	*Post-*	*Follow-up*
Baseline Knowledge, Interest, Confidence										
1	How knowledgeable are you about the problems and challenges with student success in science, technology, engineering and math (STEM)? 1- Not at all knowledgeable 2- Somewhat knowledgeable 3- Knowledgeable 4- Very knowledgeable	x			x			x		
2	How interested are you in learning more about the impact of ethnicity, race, and gender on interpersonal interactions and behavior patterns in the science classroom? 1- Not at all interested 2- Somewhat interested 3- Interested 4- Very interested	x			x			x		
3	How interested are you in modifying your teaching approaches in ways shown to benefit all students, but particularly those underrepresented students/URMs who typically do not excel in the traditional college science classroom and thus are the least likely to be retained in STEM majors? 1- Not at all interested 2- Somewhat interested 3- Interested 4- Very interested	x			x			x		
4	How confident are you that the verbal and nonverbal language you use in the classroom or the ways you communicate with students inside and outside of the classroom conveys a message of sensitivity about student diversity? 1- Not at all confident 2- Somewhat confident 3- Confident 4- Very confident	x			x			x		
Change in Knowledge										
5	Please rate your level of knowledge for each of the following topics. 1- Not at all knowledgeable (i.e., I am unfamiliar with the topic), 2- Somewhat knowledgeable (i.e., I have heard of the topic but could not readily explain it to someone else), 3- Knowledgeable (i.e., I have heard of the topic and could readily explain what it means to someone else), 4- Highly knowledgeable (i.e., I understand the current research on the topic and use it to inform the way I									
	Socioeconomic Status (SES)				x	x		x	x	
	First-generation Students				x	x		x	x	
	Underrepresented Minority (URM)				x	x		x	x	
	Inclusive Teaching Practices				x	x		x	x	
	Stereotype Threat				x	x		x	x	
	Implicit vs. Explicit Bias				x	x		x	x	
	Microaggressions				x	x		x	x	
	Classroom Climate				x	x		x	x	
	Academic Culture				x	x		x	x	
6	How has your knowledge about the problems and challenges with student success in STEM changed as a result of this workshop? 1- Increased, 2- Decreased, 3- Stayed the same		x			x			x	
7	Has your interest in learning more about the impact of ethnicity, race, and gender on interpersonal interactions and behavior patterns in the science classroom changed as a result of the workshop? 1- Increased, 2- Decreased, 3- Stayed the same		x			x				
8	Please rate your level of agreement with each statement: 1- Strongly Disagree, 2- Somewhat Disagree, 3- Somewhat Agree, 4- Strongly Agree									
	Overall, I felt this workshop gave me new insight about enhancing student success in the sciences.			x		x			x	
	I learned useful information at this workshop that will improve my teaching and interactions with students.			x		x			x	

	Closed-ended Questions	2015			2016			2017		
		Pre-	Post-	Follow-up	Pre-	Post-	Follow-up	Pre-	Post-	Follow-up
	Change in Faculty Attitudes									
9	Please rate your level of agreement with each statement: 1- Strongly Disagree, 2- Somewhat Disagree, 3- Somewhat Agree, 4- Strongly Agree									
	I recognize that not all students come into my classroom with the same level of preparedness; it is my job to help level the playing field.	x			x	x		x	x	
	Some students might perform better in my class if I used a different teaching style.	x			x	x		x	x	
	All students are capable; it is my job as their instructor to ensure that all students have equal opportunity to succeed in my class.	x			x	x		x	x	
	Some undergraduates are not cut out to be science majors and should be encouraged to leave the major as early as possible.	x			x	x		x	x	
	Change in Teaching Practices									
10	Has your interest in modifying your teaching approaches in ways shown to benefit all students, but particularly those underrepresented students who typically do not excel in the traditional college science classroom and thus are the least likely to be retained in STEM majors, changed as a result of the workshop? 1- Increased, 2- Decreased, 3- Stayed the same		x			x				
	Additional Workshop Benefits									
11	Please indicate your overall/current level of satisfaction with this workshop. 1- Very dissatisfied, 2- Somewhat dissatisfied, 3- Somewhat satisfied, 4- Very/Completely satisfied		x			x			x	
12	Please check your level of agreement with each statement: 1- Strongly Disagree, 2- Somewhat Disagree, 3- Somewhat Agree, 4- Strongly Agree									
	I would recommend this workshop to my colleagues.			x		x			x	
	The workshop sessions were well facilitated and engaged participants in useful discussions		x			x			x	
	The facilitator created a safe environment for open and productive discussions.			x		x			x	
	The opportunity to meet new people in the sciences was a positive aspect of the workshop.			x		x			x	

	Open-ended Questions	2015			2016			2017		
		Pre-	Post-	Follow-up	Pre-	Post-	Follow-up	Pre-	Post-	Follow-up
	Baseline Knowledge, Interest, Confidence									
1	What do you expect to take away from this workshop at its conclusion?	x			x			x		
	Change in Teaching Practices									
2[a]	How will you use or apply what you have learned when you return to campus?		x							
2[b]	Give an example of something you learned from this workshop and how you will use it in your teaching.					x			x	

[a,b] Letters denote minor differences in the prompt used for the same question in the surveys from year to year

	Prompt Given During Informal Group Discussion	2015	2016*	2017**
	Change in Teaching Practices			
1	Please give some things that you have incorporated into your teaching that you think were catalyzed as a result of the weekend workshop that you attended.		x	x

* Participants from years 2015/16 workshops attended group discussion in 2016; ** Participants from years 2016/17 workshops attended group discussion in 2017
