# Supplementary material for: Creating inclusive classrooms by engaging STEM faculty in culturally responsive teaching workshops
Source: Int J STEM Educ. 2020 Jul 1;7(1):32. doi: 10.1186/s40594-020-00230-7 (PMC7326892; doi:10.1186/s40594-020-00230-7)
Supplement: Supplementary file 2 — Additional file 2. Histograms associated with Figure 1. [file 40594_2020_230_MOESM2_ESM.pdf]

Creating Inclusive Classrooms by Engaging STEM Faculty in Culturally Responsive Teaching Workshops

Additional File 2. Histograms associated with Figure 1

Factor: Social Identity Awareness

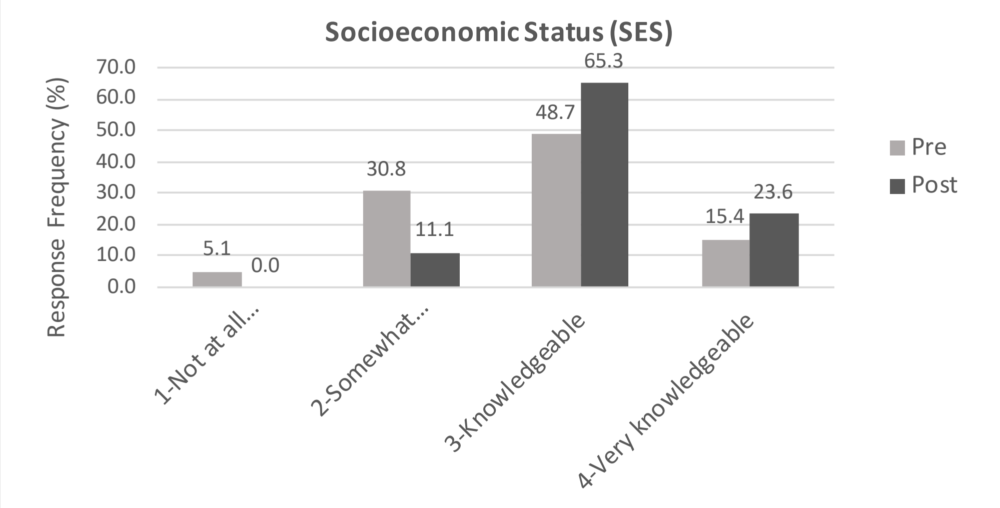

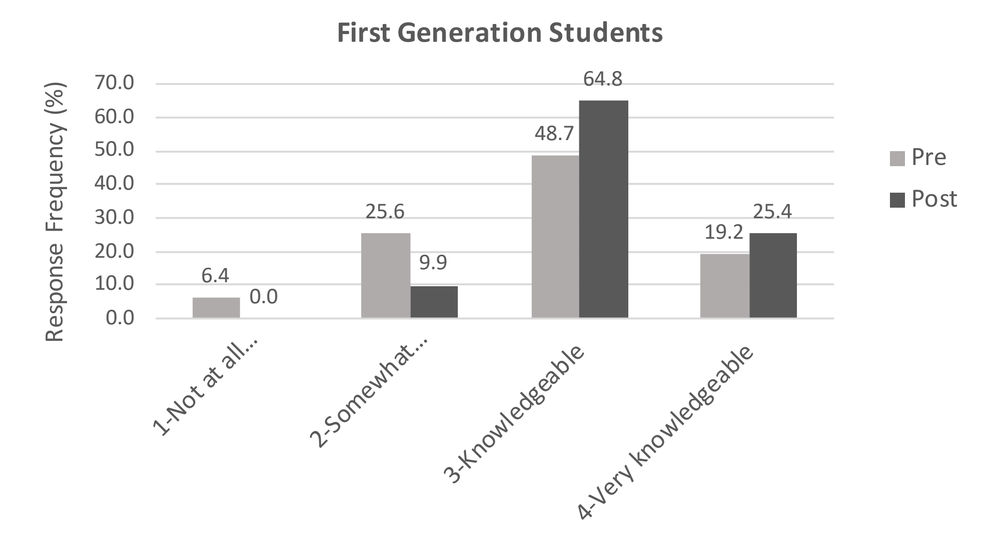

Socioeconomic Status (SES)

	Pre	Post
1-Not at all knowledgeable	5.1	0.0
2-Somewhat knowledgeable	30.8	11.1
3-Knowledgeable	48.7	65.3
4-Very knowledgeable	15.4	23.6
Mean score	2.74	3.13
SD	0.78	0.58

First-generation Students

	Pre	Post
1-Not at all knowledgeable	6.4	0.0
2-Somewhat knowledgeable	25.6	9.9
3-Knowledgeable	48.7	64.8
4-Very knowledgeable	19.2	25.4
Mean score	2.80	3.15
SD	0.82	0.58

Factor: Social Identity Awareness

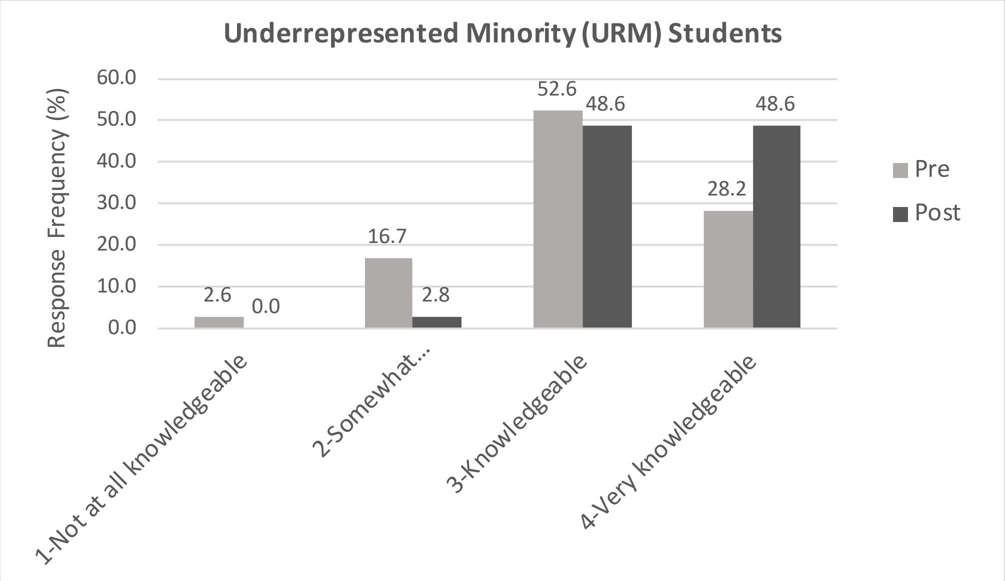

Underrepresented Minority (URM) Students

	Pre	Post
1-Not at all knowledgeable	2.6	0.0
2-Somewhat knowledgeable	16.7	2.8
3-Knowledgeable	52.6	48.6
4-Very knowledgeable	28.2	48.6
Mean score	3.06	3.46
SD	0.74	0.56

Factor: Barriers to Student Success

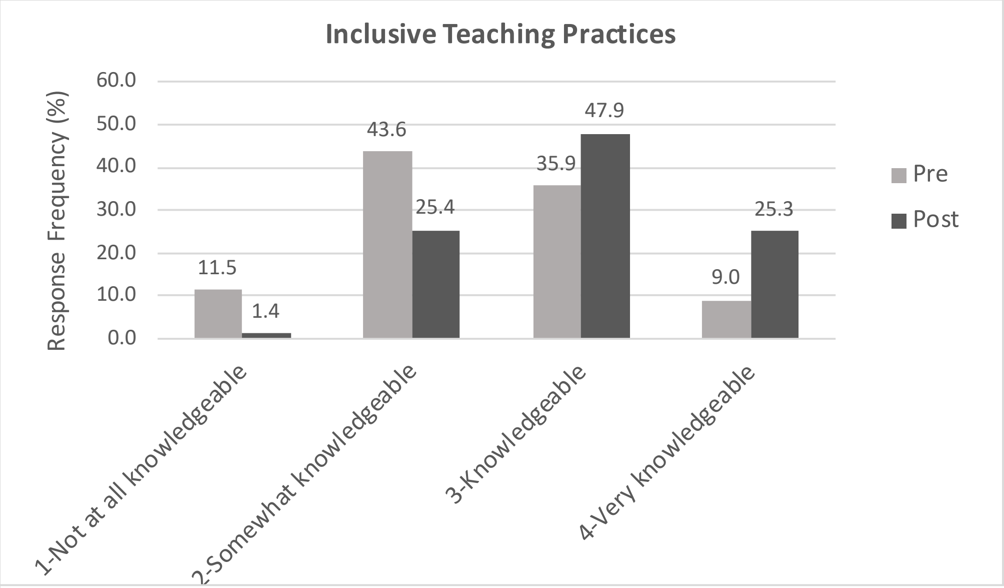

Inclusive Teaching Practices

	Pre	Post
1-Not at all knowledgeable	11.5	1.4
2-Somewhat knowledgeable	43.6	25.4
3-Knowledgeable	35.9	47.9
4-Very knowledgeable	9.0	25.3
Mean score	2.41	3.21
SD	0.81	0.58

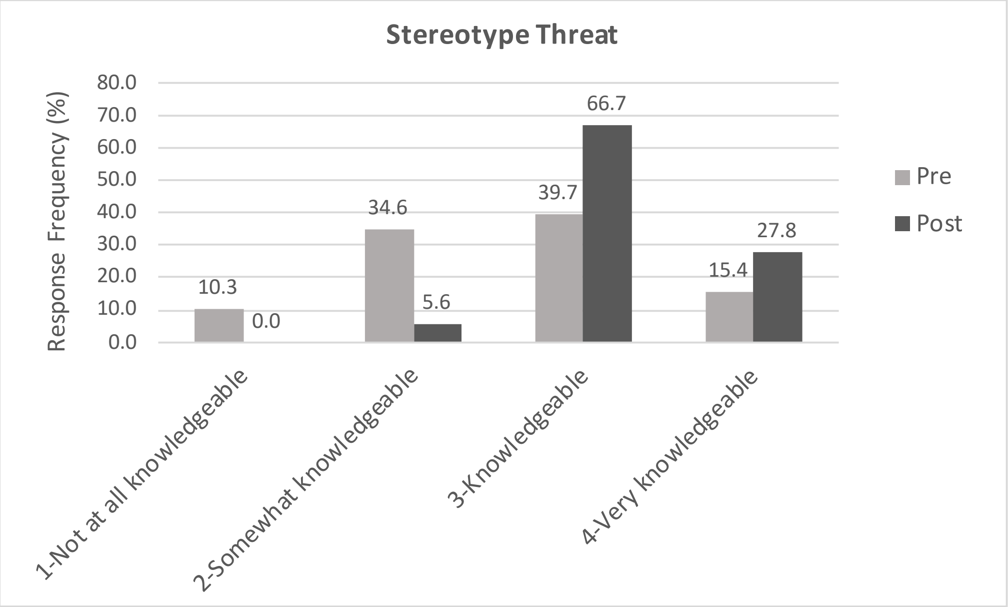

Stereotype Threat

	Pre	Post
1-Not at all knowledgeable	10.3	0.0
2-Somewhat knowledgeable	34.6	5.6
3-Knowledgeable	39.7	66.7
4-Very knowledgeable	15.4	27.8
Mean score	2.60	3.22
SD	0.87	0.54

Factor: Barriers to Student Success

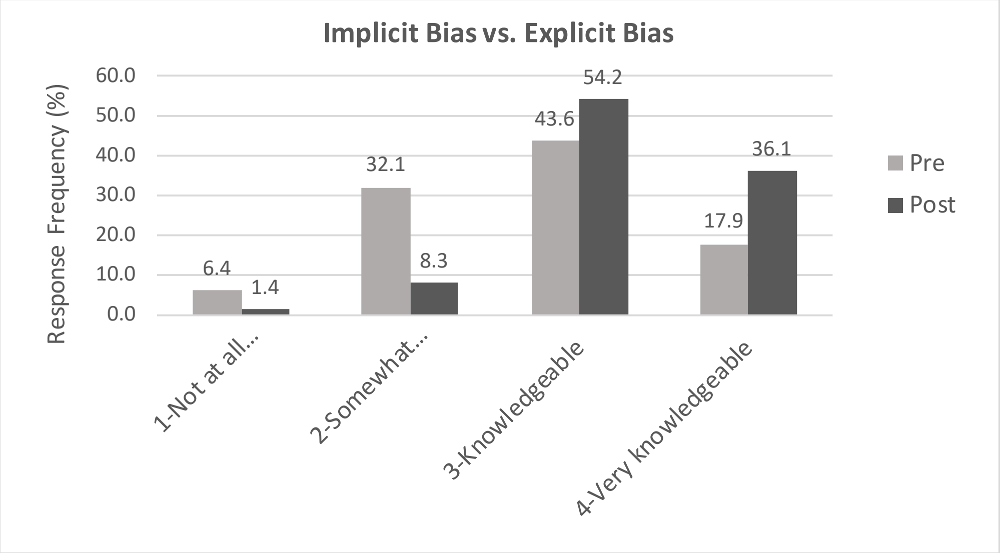

Implicit Bias vs. Explicit Bias

	Pre	Post
1-Not at all knowledgeable	6.4	1.4
2-Somewhat knowledgeable	32.1	8.3
3-Knowledgeable	43.6	54.2
4-Very knowledgeable	17.9	36.1
Mean score	2.73	3.25
SD	0.83	0.67

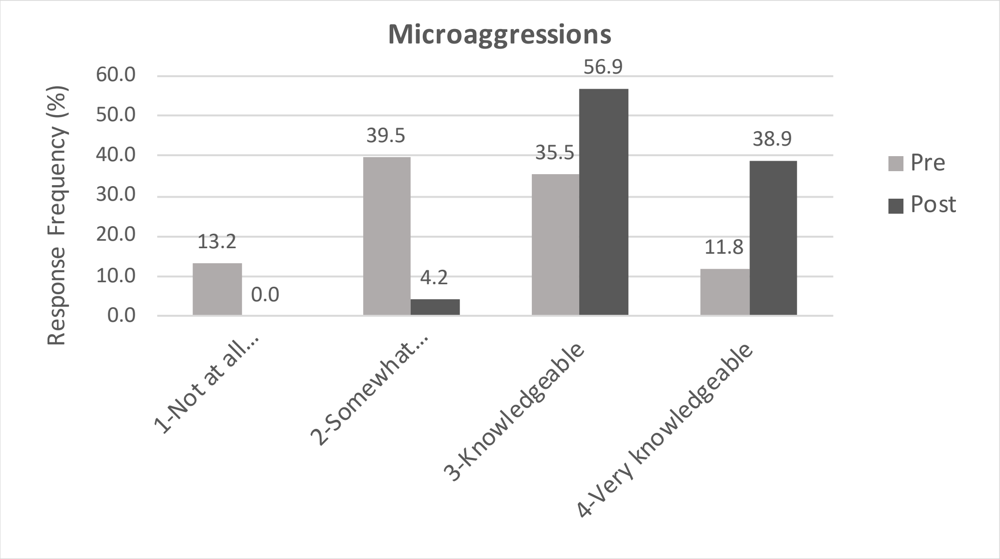

Microaggressions

	Pre	Post
1-Not at all knowledgeable	13.2	0.0
2-Somewhat knowledgeable	39.5	4.2
3-Knowledgeable	35.5	56.9
4-Very knowledgeable	11.8	38.9
Mean score	2.46	3.35
SD	0.87	0.56

Factor: Barriers to Student Success

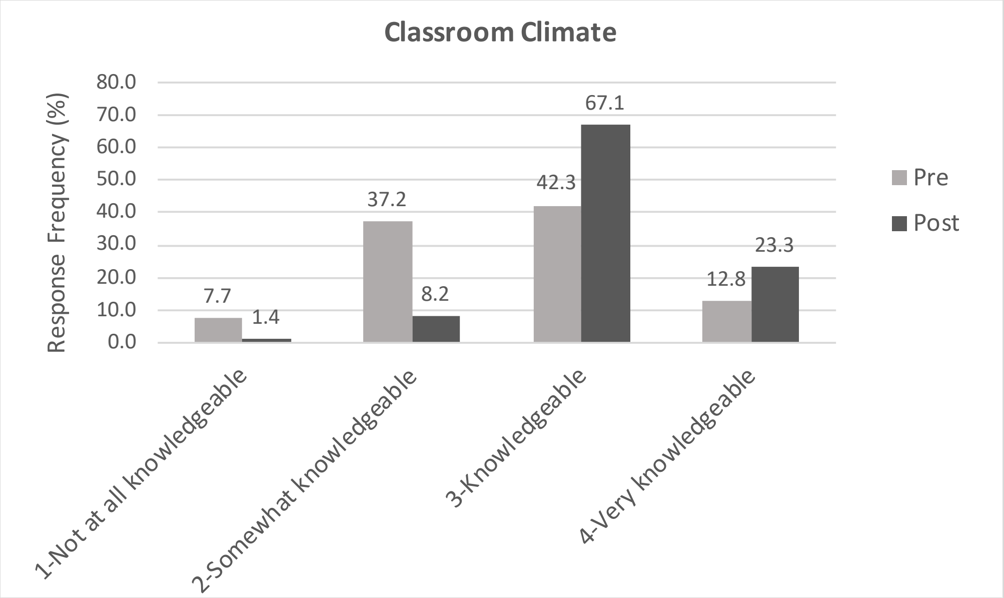

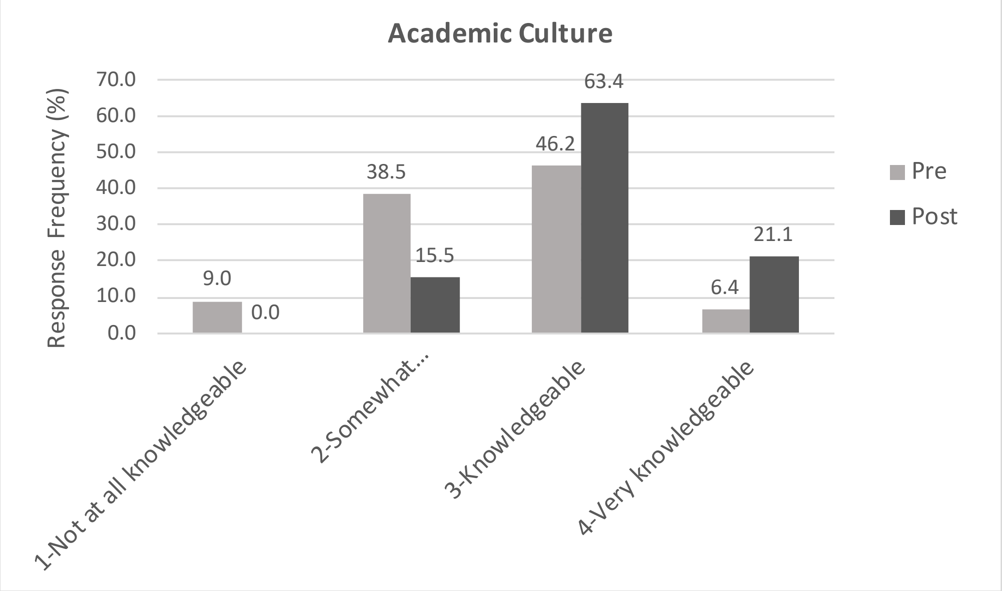

Classroom Climate

	Pre	Post
1-Not at all knowledgeable	7.7	1.4
2-Somewhat knowledgeable	37.2	8.2
3-Knowledgeable	42.3	67.1
4-Very knowledgeable	12.8	23.3
Mean score	2.60	3.12
SD	0.81	0.60

Academic Culture

	Pre	Post
1-Not at all knowledgeable	9.0	0.0
2-Somewhat knowledgeable	38.5	15.5
3-Knowledgeable	46.2	63.4
4-Very knowledgeable	6.4	21.1
Mean score	2.49	3.06
SD	0.75	0.61
