# Supplementary material for: Creating inclusive classrooms by engaging STEM faculty in culturally responsive teaching workshops
Source: Int J STEM Educ. 2020 Jul 1;7(1):32. doi: 10.1186/s40594-020-00230-7 (PMC7326892; doi:10.1186/s40594-020-00230-7)
Supplement: Supplementary file 3 — Additional file 3. Histograms associated with Table 5. [file 40594_2020_230_MOESM3_ESM.pdf]

Creating Inclusive Classrooms by Engaging STEM Faculty in Culturally Responsive Teaching Workshops

Additional File 3. Histograms associated with Table 5

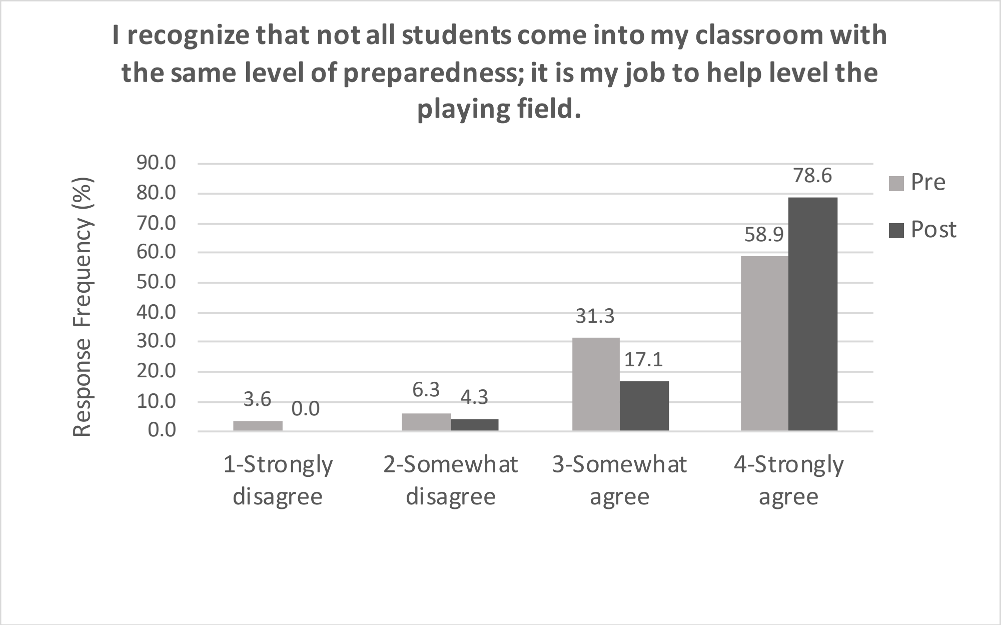

Statement: I recognize that not all students come into my classroom with the same level of preparedness; it is my job to help

	Pre	Post
1-Strongly disagree	3.6	0.0
2-Somewhat disagree	6.3	4.3
3-Somewhat agree	31.3	17.1
4-Strongly agree	58.9	78.6
Mean score	3.47	3.74
SD	0.74	0.53
n	110	70

Attitudes

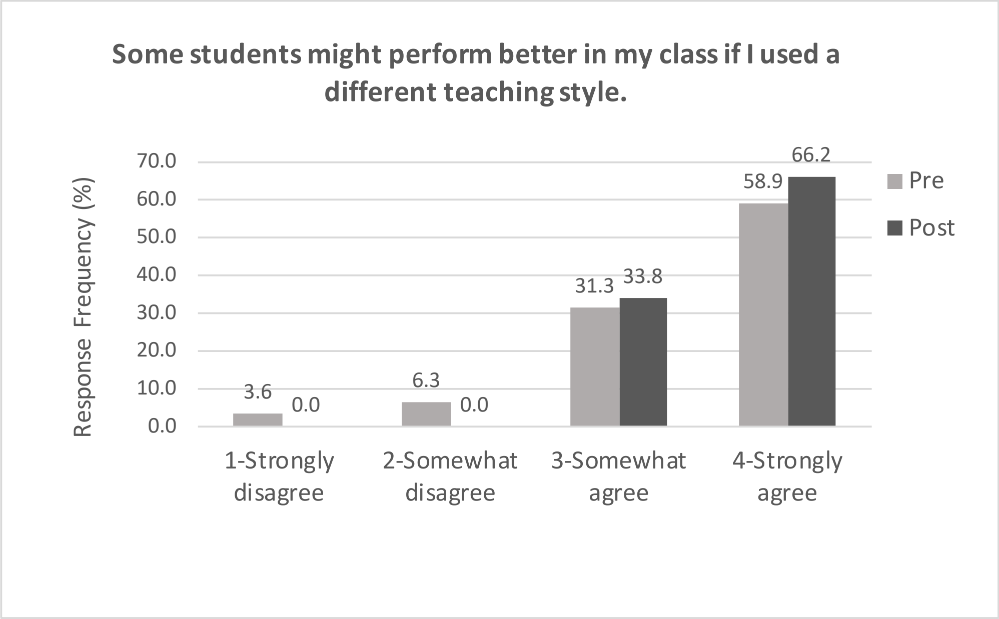

Statement: Some students might perform better in my class if I used a different teaching style.

	Pre	Post
1-Strongly disagree	3.6	0.0
2-Somewhat disagree	6.3	0.0
3-Somewhat agree	31.3	33.8
4-Strongly agree	58.9	66.2
Mean score	3.41	3.66
SD	0.66	0.48
n	113	71

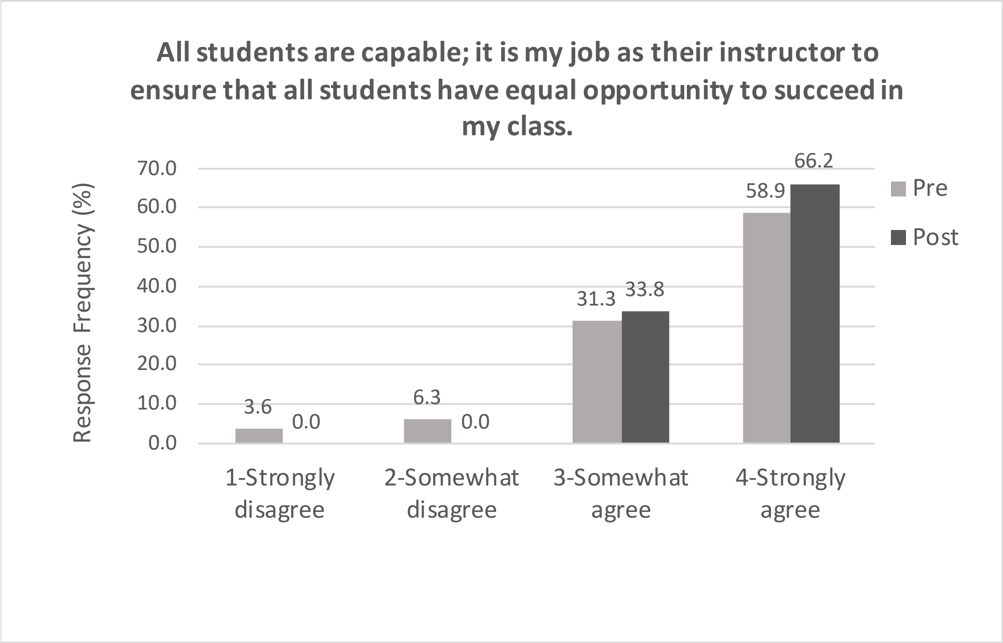

Statement: All students are capable; it is my job as their instructor to ensure that all students have equal opportunity to succeed in my class.

	Pre	Post
1-Strongly disagree	3.5	1.4
2-Somewhat disagree	7.0	2.8
3-Somewhat agree	25.4	14.1
4-Strongly agree	64.0	81.7
Mean score	3.50	3.76
SD	0.78	0.57
n	113	71

Attitudes

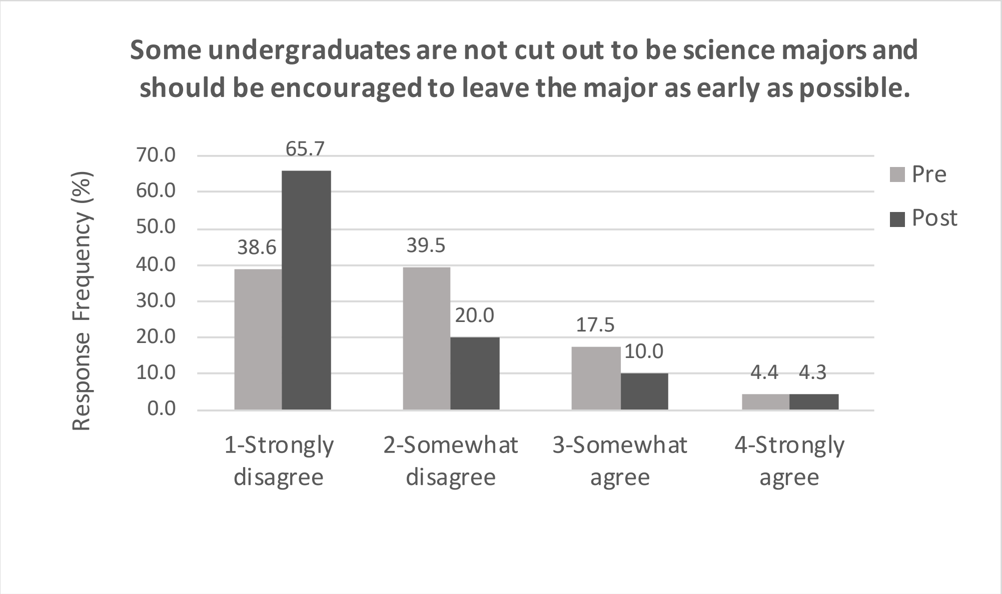

Statement: Some undergraduates are not cut out to be science majors and should be encouraged to leave the major as early as

	Pre	Post
1-Strongly disagree	38.6	65.7
2-Somewhat disagree	39.5	20.0
3-Somewhat agree	17.5	10.0
4-Strongly agree	4.4	4.3
Mean score	1.88	1.53
SD	0.86	0.85
n	112	70
